# *Himatiichnus mangano* igen. et isp. nov., a scalidophoran trace fossil from the late Ediacaran of Namibia

**DOI:** 10.1098/rsos.240452

**Published:** 2024-10-16

**Authors:** Katherine A. Turk, Mikaela A. Pulsipher, Helke Mocke, Marc Laflamme, Simon A. F. Darroch

**Affiliations:** ^1^ Department of Earth and Environmental Sciences, Vanderbilt University, Nashville, TN, USA; ^2^ Evolutionary Studies Institute, Vanderbilt University, Nashville, TN, USA; ^3^ Department of Geological Sciences, University of Missouri, Columbia, MO, USA; ^4^ Grand Canyon National Park, National Park Service, Flagstaff, AZ, USA; ^5^ Geological Survey of Namibia, Ministry of Mines and Energy, Windhoek, Namibia; ^6^ University of Toronto Mississauga, Mississauga, Ontario, Canada; ^7^ Senckenberg Museum of Natural History, Frankfurt 60325, Germany

**Keywords:** ichnology, Ediacaran–Cambrian transition, Nama Group, Scalidophora, Priapulida, Palaeoscolecida

## Abstract

*Himatiichnus mangano* igen. et isp. nov., a new trace fossil from the late Ediacaran Huns Member of the Urusis Formation, southern Namibia, comprises intertwining tubes exhibiting dual lineation patterns and reminiscent of both modern and early Cambrian examples of priapulid worm burrows. These similarities support the interpretation of a total-group scalidophoran tracemaker for *H. mangano*, thus providing direct evidence for the first appearance date of Scalidophora in the late Ediacaran *ca* 539 Ma. This new material is thus indicative of the presence of total-group scalidophorans below the Cambrian boundary and supports inference of a lengthy Precambrian fuse for the Cambrian explosion.

## 1. Introduction

The terminal Ediacaran ‘Nama’ assemblage approximately 550–538 Ma [1–3] is a key interval in the history of life, preserving both soft-bodied Ediacara biota (an enigmatic collection of soft-bodied organisms with uncertain relationships to extant animal phyla) and a burgeoning fauna of more recognizable animals reminiscent of the Cambrian [4–9]. This interval is thus increasingly thought to record a ‘long fuse’ for the Cambrian explosion and is key to understanding the origins of the modern animal-dominated marine biosphere [5,7,10–15].

While evidence from molecular clocks suggests Ediacaran divergence times for many animal taxa [16,17], there remains a lack of definitive fossil evidence for many of these groups from below the Ediacaran–Cambrian boundary (ECB). Of particular interest are the Ecdysozoa—a clade united by the common process of cuticular moulting—and comprises the Panarthropoda (Arthropoda, Onychophora and Tardigrada), Nematoida (Nematoda and Nematomorpha) and Scalidophora (Kinorhyncha, Loricifera and Priapulida) [18–20]. Ecdysozoa represents the vast majority of both extant and fossil animal diversity, with an estimated 5.5 million living species [21,22]. The recent discovery of putative ecdysozoans from the late Ediacaran of China [23] aside, the earliest indisputable ecdysozoan body fossils are known from the Fortunian [24], and the ichnofossil *Treptichnus pedum*—interpreted as the repeated arcuate probing of a priapulan-grade organism [25]—is used as a biostratigraphic marker for the base of the Cambrian [26,27]. However, divergence time estimation analyses [20] suggest crown-group ecdysozoans first appeared between 636 and 578 Ma, thus indicating a substantial missing Ediacaran fossil record for this group.

Within Ecdysozoa, priapulids and palaeoscolecids represent two groups that are potentially crucial to understanding the character of benthic ecosystems over the Ediacaran–Cambrian transition. Priapulid worms (Cambrian–recent) are scalidophorans distinguishable by their evaginable frontal introvert possessing rings of equally spaced protruding scalids. This introvert transitions into an annulated trunk and often one or two caudal appendages thought to serve respiratory and/or defensive functions [28–32]. Priapulids are common components of Cambrian assemblages [33–36], although they are today represented by a comparatively sparse 19 species largely restricted to high-latitude, poorly oxygenated muddy sediments [25,37–39]. Traces attributed to priapulids are also notable, with the aforementioned *T. pedum* used to mark the ECB and well-preserved ichnofossils known from the Lower Cambrian of Sweden [40].

By contrast, palaeoscolecids (Cambrian–Silurian) are worm-like animals bearing an annulated cuticle possessing phosphatic or phosphatized arrays of transverse sclerites and are often interpreted as scalidophorans, although their exact taxonomic placement within the Ecdysozoa remains a subject of debate [34,41–44]. Palaeoscolecids represent the most diverse subset of fossil record scalidophorans, with more than 60 species (45 genera) described; one-third of palaeoscolecid genera have been described on the basis of microscopic cuticle and scleritome fragments alone [44]. Palaeoscolecids have been posited as major sediment bioturbators [45], although their endobenthic life habits have largely been inferred based on morphological features such as aboral hooks, cylindrical bodies and radial sclerite distribution [34,46–48].

Here, we describe a new trace fossil attributable to vermiform scalidophorans such as priapulids or palaeoscolecids from the late Ediacaran Huns Member (Urusis Formation) of the Nama Group, with broad implications for understanding late Ediacaran palaeoecology and the early history of a key extant animal phylum.

## 2. Geologic setting

**Figure 1.**
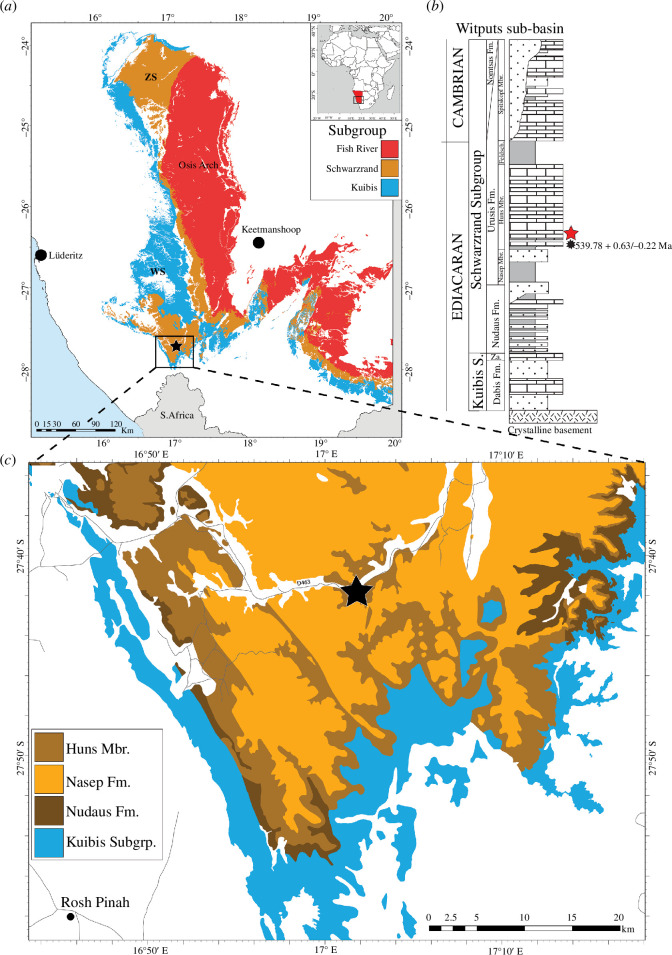
(*a*) Map of Nama Group sediments in Namibia south of Windhoek; Zaris (ZS) and Witputs (WS) sub-basins are indicated (modified from [6]); shapefiles are sourced from the Geological Survey of Namibia. (*b*) Generalized stratigraphy of the Witputs sub-basin, with star indicating position of Farm Arimas (adapted from [6]; dates follow from [3]). (*c*) Close view of the study area within the Witputs; star indicates the location of Farm Arimas.

The Ediacaran–Cambrian Nama Group of southern Namibia consists of more than 3000 m of mixed siliciclastic-carbonate sedimentary rocks deposited in a foreland basin formed due to orogenic activity associated with the assembly of Gondwana (figure 1) [49–55]. South of Windhoek, the Nama is subdivided into two sub-basins (the northern Zaris and southern Witputs) partitioned by the central high Osis Arch [49,55].

Within the Witputs Sub-basin, the Urusis Formation is subdivided into the Nasep, Huns, Feldschuhhorn and Spitskop members [55,56]. The Nasep–Huns transition is interpreted as representing a transgressive sequence, with the upper Nasep sandstone interpreted as a delta slope-to-coastal plain depositional environment [55–57]. The overlying Huns limestone, by contrast, represents a storm-dominated carbonate ramp succession with an inner ramp, ramp crest and ramp-to-basin transition facies recognized in various parts of the basin [53,55–57]. The Huns Member primarily comprises thin stromatolitic intervals, cross-stratified limestone grainstone and interspersed limestone intraclast conglomerate, with the lower Huns (0–40 m) characterized by shale–limestone interbeds that transition into metre-scale stromatolitic units and small patch reefs in the upper approximately 260 m [56,58].

The position of the ECB in the Nama Group is currently disputed. Traditionally, the boundary has been placed stratigraphically between the Urusis and Nomtsas Formations, which in many localities is marked by an erosive unconformity [49,53,55,58–60]. While some studies have argued that the ECB lies toward the top of the Spitskop Member [61], recent work on Nama-equivalent strata from the nearby Neint Nababeep Plateau in South Africa suggests that much of the Nomtsas Formation may also be Ediacaran, and thus, the ECB is stratigraphically higher [3]. In all current models, however, the Huns Member is interpreted as Ediacaran, underlying dense accumulations of erniettomorph Ediacara biota in the Spitskop Member and below the last appearance dates of *Cloudina* and *Namacalathus* [59,61–63]. Employing a Bayesian age-depth model, the Nasep–Huns contact and, thus, the approximate stratigraphic position of the trace fossil material described here can be precisely constrained to 539.78 +0.63/−0.22 Ma (see electronic supplementary material, table S3 in [3]).

## 3. Description of new trace material

*Institutional abbreviations.* Geological Survey of Namibia (GSN), Windhoek, Namibia.

            Ichnogenus *Himatiichnus* igen. nov.

*Type and only ichnospecies. Himatiichnus mangano* isp. nov.

*Diagnosis.* Complex trace fossil comprising non-tapering, meandering, longitudinally striated tubes, with rare faint transverse annulations in the medial portion. In some instances, tubes appear to dip below and re-emerge from the sediment surface and terminate with rounded, bulbous structure(s).

*Etymology.* From the ancient Greek garment *himation*, the draped parallel folds of which are visually similar to the longitudinal striations present on this material.

*Himatiichnus mangano* isp. nov.

2022 Unnamed longitudinally striated traces; Turk *et al*. [57], fig. 9.

**Figure 2.**
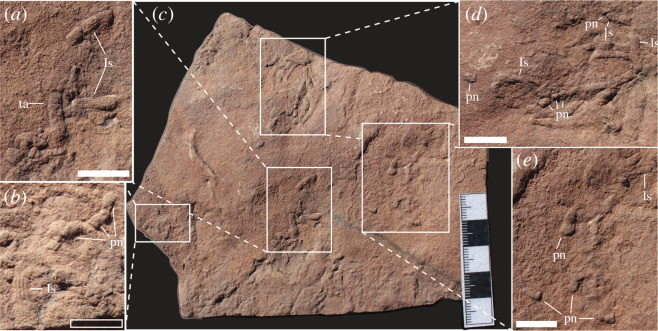
(*a*) Holotype specimen of *H. mangano*, probably representing bed sole. (*b,d,e*) Additional examples of *H. mangano*. (*c*) Full slab (GSN F1643). Abbreviations: ls, longitudinal striations; ta, transverse annulations; pn, probing nubs. Filled scale bars in (*a*), (*d*), (*e*) = 1 cm, hollow in (*b*) = 5 mm.

*Holotype.* Specimen A preserved on GSN F1643 (figure 2*a*).

*Diagnosis*. As for ichnogenus.

*Etymology.* In recognition of the significant contributions of Dr. M. Gabriela Mángano to ichnology and our understanding of the Ediacaran–Cambrian transition.

*Material and stratigraphic setting*. One slab (GSN F1643) with four prominent specimens; specimen A (figure 2*a*) is denoted as the holotype. These traces were recovered from the lower Huns Member of the Urusis Formation at Farm Arimas (27°41′36.1″ S, 17°1′50.5″ E), approximately 55 km west of the Fish River Canyon. The material is reposited at the GSN in Windhoek.

Traces are preserved in convex semi-relief (probably from bed sole) on reddish sandstone collected from an interval of subcrop (approx. 78 m above the lowermost exposures at Arimas) also preserving examples of *Corumbella*, bounded both below and above by thick- to very thick-bedded sandy limestone.

*Description*. Intertwining 1–3 cm long tubes (approx. 2–3 mm in width) preserved in convex semi-relief and exhibiting consistently spaced longitudinal striations approximately 0.4 mm apart; in some instances, this patternation shifts to a transverse annulation in the more proximal (i.e. closer to the point of radiation) portions of the material. Individual tubes are 2–3 cm in width and appear in several instances to disappear beneath and re-emerge on top of the sediment surface. Structures maintain constant width with no evidence of terminal tapering; distal ends (furthest from the point of radiation) terminate in a rounded, bulbous manner. Some distal portions preserve a series of nub-like protrusions (figure 2*b*,*d*) immediately adjacent to burrow terminations.

**Figure 3.**
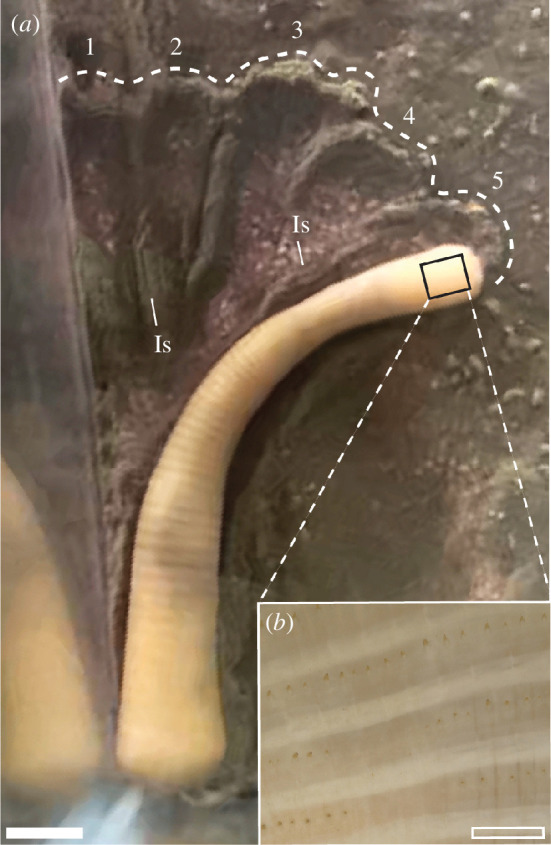
(*a*) Specimen of *P. caudatus* exhibiting surficial probe–retract–probe behaviour; individual probes marked with numbers, where 1 indicates the first probe and 5 indicates the most recent. The outline of greater probing structure is marked by a dotted line. Abbreviation: ls, longitudinal striations. (*b*) Close view of introvert displaying evenly spaced scalids. Filled scale bar in (*a*) = 1 cm, hollow in (*b*) = 1 mm.

*Remarks*. *Himatiichnus mangano* is interpreted to have formed as an animal bearing convex frontal ornamentation, and anterior transverse lineation moved across—and penetrated slightly beneath—the sediment surface, while the series of nub-like structures found adjacent to the terminations suggest a repeated probing behaviour by the organism along the direction of motion. These factors are consistent with both the anatomy and burrowing behaviours of extant priapulid worms. Priapulid locomotion is a multi-step process, beginning with an evagination of the frontal introvert on which are present lines of equally spaced protrusions (scalids; figure 3*b*) that converge at the mouth [29,64]. These posteriorly oriented scalids serve to increase friction between the animal and substrate, anchoring the proboscis and thus allowing the worm to generate a peristaltic wave that shortens the annulated trunk, which then shifts into the space previously occupied by the frontal portions of the animal. Upon reaching the posterior praesoma, the muscular contractions cease, and the introvert is evaginated to begin the process anew [65]. A neoichnological study [40] demonstrated that experimentally produced casts of modern priapulid burrows bear notable resemblance to early Cambrian material described in the same paper; of particular note was the dual patternation of equally spaced longitudinal striations transitioning into transverse annulations. The multi-pronged, braided structure is indicative of repeated probe–retract–probe behaviour commonly exhibited by modern priapulids (i.e. *Priapulus caudatus*) when vertically restricted [25,66] (figure 3*a*).

## 4. Discussion

The attribution of *H. mangano* to probing traces left by vermiform scalidophorans aids our understanding of the timing and impacts of animal evolution in the late Ediacaran. We first discuss the anatomy of priapulids versus palaeoscolecids in the context of these new traces, a potential match with divergence times for these groups obtained from molecular clocks, and explore the roles these organisms and behaviours may have played in late Ediacaran communities. Lastly, we discuss the evolving picture of a transitional Nama interval comprising elements of both Ediacaran and Cambrian-type faunas, thus lending new support for a ‘long fuse’ model for the Cambrian explosion and emergence of Phanerozoic-type ecosystems.

### 4.1. Scalidophorans in the late Ediacaran

Several features seen in the *H. mangano* holotype material are suggestive of priapulid or palaeoscolecid tracemakers. Both priapulids and palaeoscolecids exhibit an overall dual surface patternation, with their anterior portions characterized by cuticular ornaments (scalids and spines, respectively). In modern macroscopic priapulids, there is no transition between the introvert and the densely annulated trunk, the morphology of which is controlled by regular spacing of circular muscle bundles along its length [39]. Palaeoscolecids also possess annulated trunks, with individual annulations marked by regularly spaced button-like sclerites along the length of the body [42,67]. The material described here as *H. mangano* displays a double patterned surface, with the distal portions (furthest from the point of tube origination) characterized by equally spaced longitudinal striations. In some examples, these appear to transition into proximal sections (closer to the tube origination point) exhibiting equidistant transverse annulations. This surface patternation closely matches the priapulid and palaeoscolecid anatomies described above, with neoichnological work [40,66] showing that the burrowing of modern priapulids produces visually similar striae as the friction-generating scalids (figure 3*b*) are dragged parallel to the overall direction of motion. The largely horizontal nature of the traces is also consistent with experimental results demonstrating that modern priapulids restricted to a single horizontal plane produce similar ‘probing’ traces to the material found here [25,66] (also see figure 3*a*).

The age constraints on these new fossils also provide a close match with divergence time estimates obtained via molecular clocks. A fossil-calibrated Bayesian analysis of protein-coding genes has obtained a divergence time estimate of 617−534 Ma for crown-group Scalidophora [20]. Using a Bayesian age-depth model, the Nasep–Huns contact and, thus, the approximate stratigraphic position of *H. mangano* can be precisely constrained to 539.78 +0.63/−0.22 Ma [3]. While trace fossils left by potential ecdysozoans are known between 551 and 555 Ma [23,68,69], *H. mangano* represents the earliest-known example of trace material with clear total-group scalidophoran affinity, especially in the context of comparison with both early Cambrian and modern priapulid traces [40,70]. Our trace fossil material thus represents an updated first appearance date (FAD) for scalidophorans and provides support for a deeper Ediacaran divergence of sister lineages of Priapulida and/or Scalidophora (i.e. total-groups Kinorhyncha, Loricifera and Cryptovermes [20]).

It is important to note, however, that the extent to which these lineages stretch into the Ediacaran is dependent on the placement of the ECB. While the current geologic time scale places the ECB at 538.8 Ma [61,71], recent high-precision geochronological work on the lower Wood Canyon Formation of Nevada has suggested that the boundary (as defined by the FAD of *T. pedum*) may be as young as 533 Ma [72]. As it currently stands, *H. mangano* precedes the ECB by approximately one million years; however, successive calibrations of the ECB over the last 40 years have indicated progressively younger dates [72]. Thus, with further refinement, it is quite possible this new material may instead represent a several million year-long antecedent to the Cambrian.

### 4.2. Late Ediacaran community palaeoecology

The new trace fossils described here form part of a diverse ichnofossil assemblage from the Nasep–Huns transition, alongside *Archaeonassa*, *Gordia*, *Helminthoidichnites*, *Helminthopsis*, *Torrowangea*, small treptichnids and meiofaunal traces [57]. Other studies [73,74] targeting this transition have also described *Curvolithus*, *Didymaulichnus*, *Palaeophycus*, *Planolites* and *Skolithos*, although these ichnotaxa have not been found by our group (and occurrences of *Skolithos* in particular are now thought to instead represent plug-shaped burrows such as *Conichnus* and *Bergaueria* [6,75]). Alongside these trace fossils, the body fossil *Corumbella werneri*—interpreted as either an early scyphozoan cnidarian [76–78] or a calcareous sinotubilitid [79]—has been reported from nearby horizons [57]. The addition of *H. mangano*—a trace fossil more easily attributed to the responsible animal tracemaker(s) than many others from this interval—paints a clearer picture of late Ediacaran ecological diversity and reinforces the inference that benthic communities preserved in the upper Schwarzrand Subgroup of the Nama Group possess many ecological features typically associated with the Cambrian.

Based on preserved morphology, the *H. mangano* tracemaker probably bore affinities with scalidophorans such as priapulids or palaeoscolecids. As an entirely extinct group, the ecology of palaeoscolecids is little understood, although preserved gut remains [47,48,80–82] indicate that Cambrian examples were carnivorous [83] and deposit feeders that often consumed smaller invertebrates such as *Isoxys* [46,48]. Priapulids, by contrast, survive into the present day, and as such, more is known about their behaviour and ecology. Priapulids are thought to have been overwhelmingly predatory since at least the early Palaeozoic [84–86]; preserved gut contents from these animals have been found to contain a variety of small marine invertebrate material, including elements from hyolithids, brachiopods, bradoriids, trilobites, agnostids, polychaetes and wixwaxiids [87]. These new trace fossils thus reinforce the inference that predatory and/or deposit-feeding behaviours were well-established by the late Ediacaran [88,89] and, thus, probably played a part in structuring benthic communities and driving successive waves of evolutionary radiation over the Cambrian explosion [13,16,90].

Lastly, the attitude and orientation of *H. mangano* traces may also offer insight into the character of organism–substrate interactions over the ECB. Despite the dramatic increase in trace fossil diversity seen in the Nama interval (relative to the older White Sea and Avalon), recent work [6,91] indicates that ichnofossil assemblages continued to be dominated by biomixing, rather than bioirrigative behaviours, and moreover, that the impact of these traces would probably have been to shallow the depth of oxygen penetration [91]. Our specimens of *H. mangano* indicate an overall burrow architecture similar to those of treptichnids (i.e. gallery-type burrows characterized by repeated offset probes beneath the sediment–water interface) but which would have been extremely shallow, probably less than 1 cm deep. This contrasts starkly with modern priapulids, which will burrow along a horizontal path (often with offset probes deviating 20°–40° from the overall direction of motion) only when vertical motion is restricted [25,66] (figure 3*a*). The shallow depth of *H. mangano* burrows may potentially reflect a barrier to subsurface movement imposed, for example, by both lower shallow marine oxygen levels and a redox discontinuity surface close to the surface and largely unaffected by the predominant biomixers [40,92], with more modern-looking deep-tier scalidophoran burrows only appearing later in the Cambrian with the emergence of more intense bioirrigators facilitating increased subsurface oxygenation [91].

## 5. Summary

We describe *H. mangano*, a new, complex trace fossil from low in the Ediacaran Huns Member of the Nama Group in southern Namibia, characterized by repeated probes possessing both longitudinal and transverse lineations. We interpret these burrows as being formed by scalidophorans such as priapulids or palaeoscolecids, with longitudinal striations produced by a circular arrangement of frontal extrusions as they are pushed through the sediment, and transverse structures representing the preserved impressions of an annulated trunk. The late Ediacaran horizons preserving these traces are constrained to precisely 539.78 +0.63/−0.22 Ma, representing an updated first appearance date for total-group Scalidophora and providing support for a deeper Ediacaran divergence of Ecdysozoa. These burrows also contribute to an emerging picture of Nama-aged Ediacaran ecosystems that is far more similar to those of the lower Cambrian than previously appreciated, with a diversity of metazoan clades present and exhibiting a variety of predatory, deposit-feeding and suspension-feeding lifestyles. In turn, these late Ediacaran communities contribute to a burgeoning weight of support for the ‘long fuse’ model for the Cambrian explosion, illustrating that organisms, behaviours and ecological interactions once thought to be confined to the Palaeozoic now extend millions of years back into the late Ediacaran.

## Data Availability

The specimens used as the basis for this study are accessioned at the Geological Survey of Namibia in Windhoek; accession number: GSN F1643.
